# Evaluation of Controlled Ovarian Stimulation Protocols in Patients with Normal and Low Ovarian Reserve: Analyses of miRNAs and Selected Target Genes Involved in the Proliferation of Human Cumulus Cells and Oocyte Quality

**DOI:** 10.3390/ijms23031713

**Published:** 2022-02-02

**Authors:** Giulia Russo, Valentina Notarstefano, Nina Montik, Giorgia Gioacchini, Elisabetta Giorgini, Anna Rita Polidori, Fulvia Antonia Candela, Andrea Ciavattini, Maurizio Cignitti, Oliana Carnevali

**Affiliations:** 1 Department of Life and Environmental Sciences, Università Politecnica delle Marche, Via Brecce Bianche, 60131 Ancona, Italy; giu-rus@hotmail.com (G.R.); v.notarstefano@staff.univpm.it (V.N.); giorgia.gioacchni@staff.univpm.it (G.G.); e.giorgini@staff.univpm.it (E.G.); 2 Clinica Ostetrica Ginecologica, Università Politecnica delle Marche, Ospedale G. Salesi, Via F. Corridoni 11, 60131 Ancona, Italy; nina.montik@ospedaliriuniti.marche.it (N.M.); annarita.polidori@ospedaliriuniti.marche.it (A.R.P.); fulviaantonia.candela@ospedaliriuniti.marche.it (F.A.C.); andrea.ciavattini@staff.univpm.it (A.C.); maurizio.cignitti@ospedaliriuniti.marche.it (M.C.)

**Keywords:** rFSH treatment, hMG treatment, oocyte quality, ovarian reserve, cumulus cells, assisted reproduction technology (ART), DNA conformation, FTIR microspectroscopy

## Abstract

The oocyte and the surrounding cumulus cells (CCs) are deeply linked by a complex bidirectional cross-talk. In this light, the molecular analysis of the CCs is nowadays considered to be precious in providing information on oocyte quality. It is now clear that miRNAs play a key role in several ovarian functions, such as folliculogenesis, steroidogenesis, and ovulation. Thus, in this study, specific miRNAs, together with their target genes, were selected and investigated in CCs to assess the response of patients with normal (NR) and low (LR) ovarian reserve to two different controlled ovarian stimulation (COS) protocols, based on rFSH and hMG. Moreover, a Fourier transform infrared microspectroscopy (FTIRM) analysis was performed to evaluate DNA conformational changes in CCs and to relate them with the two COS protocols. The results evidenced a modulation of the expression of miRNAs and related target genes involved in CCs’ proliferation, in vasculogenesis, angiogenesis, genomic integrity, and oocyte quality, with different effects according to the ovarian reserve of patients. Moreover, the COS protocols determined differences in DNA conformation and the methylation state. In particular, the results clearly showed that treatment with rFSH is the most appropriate in NR patients with normal ovarian reserve, while treatment with hMG appears to be the most suitable in LR patients with low ovarian reserve.

## 1. Introduction

A complex bidirectional communication is observed between the oocyte and the cumulus cells (CCs) that surround it, which not only have trophic functions, but are also involved in the maintenance of the meiotic block of the oocyte during follicular growth [1]. The study of the cumulus-oocyte complex allows one to establish that a ‘competent’ oocyte cannot exist without a ‘competent’ cumulus. For this reason, a molecular analysis of the CCs is nowadays considered predictive of oocyte quality [2]. For example, the stage of CC development is associated with the fate of the follicle: their proliferation promotes follicle maturation and ovulation, while their apoptosis causes atresia and follicle degradation [3]. In this light, for proper follicular recruitment, development, and ovulation, somatic and germinal cells within the ovaries need to precisely express specific genes in a coordinated fashion [4]. MicroRNAs (miRNAs) represent a class of small (18–22 nt) non-coding, single-stranded RNAs actively regulating the mRNA translation of over 70% of protein-coding genes, and they are widely involved in both normal and pathological states [5]. MiRNAs’ role in ovarian cells has been partially elucidated, and their influence on key ovarian functions such as steroidogenesis, ovulation, and the development and function of the corpus luteum has been discovered [6]. Recent evidence showed the regulatory function of miRNAs in oocyte maturation and ovarian follicular development by promoting granulosa cells proliferation [7,8,9,10], or cell apoptosis and the formation of the corpus luteum [11,12,13,14]. Thus, understanding the mechanisms underlying the way in which somatic cell miRNAs are regulated and identifying their specific target genes and functions can lead to the development of strategies to achieve specific gene regulation for the prevention and treatment of various ovarian disorders.

Since in ART there is always a need to improve the knowledge of key molecules associated with the development of the embryo, in order to reach advanced diagnostic practices, follicular cells’ miRNAs have recently been taken into account as potential reliable biomarkers in level II (in vitro fertilization and embryo transfer, IVF-ET, and intracytoplasmic sperm injection, ICSI) and III ART (testicular sperm aspiration, TESA, testicular sperm extraction, TESE, followed by ICSI) techniques.

In ART practices, over the years, in parallel with the introduction of increasingly targeted and advanced drugs, new protocols and procedures for the personalization of treatments have been developed in order to offer women ovarian stimulation techniques increasingly suited to their individual profile. In the management of infertility, the main hormonal biomarker is the anti-Müllerian hormone (AMH), used as a direct measure of the ovarian state, in order to personalize the treatment as much as possible. According to the European Society of Human Reproduction and Embryology (ESHRE) guidelines (2019), during ovarian stimulation for IVF/ICSI, the use of antral follicle count (AFC) or the quantification of anti-Müllerian hormone (AMH) levels is recommended compared to other ovarian reserve tests [15,16]. Although there is no unanimously recognized cut-off, due to the different quantification techniques of this hormone, to stratify patients based on ovarian reserve, in this study, we divided the cohort of patients into patients with low reserve, LR (AMH < 1.2 ng/mL) [17], and normal reserve, NR (AMH > 1.2–4.0 ng/mL) [18].

Nowadays, several strategies are available to induce ovarian stimulation that differs in the type and dose of gonadotropins administered, and in the mode of suppression of the pituitary gland [19]. An ideal stimulation protocol should reduce risks and side effects, allow for a low cycle cancellation rate, and involve limited endocrine and ultrasound control. Unfortunately, to date, a single protocol capable of possessing all these characteristics has not yet been developed: the advantages and disadvantages must be assessed individually, based on certain characteristics, such as the woman’s age, ovarian reserve, and response. One of the most exploited controlled ovarian stimulations (COS) protocols includes treatment with follicle-stimulating gonadotropins, which can be recombinant, indeed synthesized, through genetic engineering techniques (e.g., follitropin α, follitropin β, corifollitropin α, and lutropin α), or menopausal gonadotropins, hence extracted from the urine of postmenopausal women, containing FSH and LH in different proportions (menotropin, or hMG), or containing only FSH (urofollitropin).

**Figure 1:**
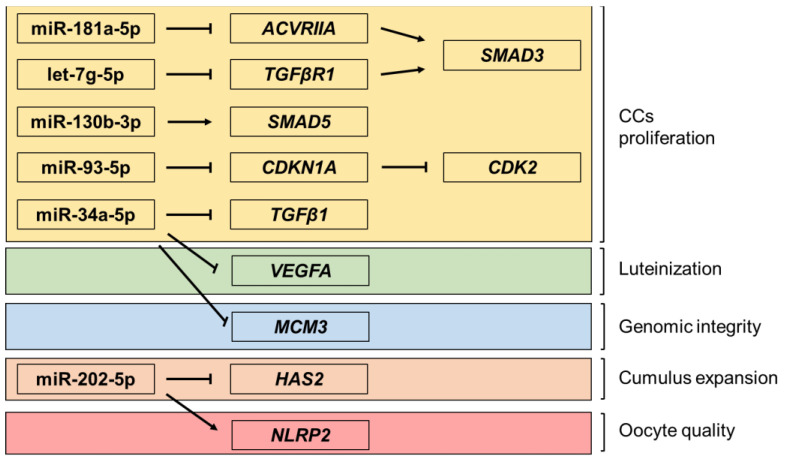
Scheme displaying the miRNAs selected in the study, together with their related genes and the pathways they are involved in. *ACVRIIA*: activin A receptor type 2A; *SMAD3*: smad family member 3; *TGFβR1*: transforming growth factor beta receptor 1; *SMAD5*: smad family member 5; *CDKN1A*: cyclin dependent kinase inhibitor 1A; *CDK2*: cyclin dependent kinase 2; *TGFβ1*: transforming growth factor beta 1; *VEGFA*: vascular endothelial growth factor; *MCM3* minichromosome maintenance complex component 3; *HAS2*: hyaluronan synthase; *NLRP2:* NLR family pyrin domain containing 2.

The aim of the present study was to elucidate the different responses of low and normal ovarian reserve patients to different ovarian stimulation protocols (recombinant FSH, rFSH, and human menopausal gonadotropin, hMG), on the expression of selected miRNA and their target genes, in CCs retrieved by ART patients with normal and low reserves. In this study, the following miRNAs were considered: miR-181a-5p, miR-93-5p, miR-130b-3p, let-7g-5p, and miR-34a-5p. Moreover, the expression of some of their target genes, involved in the regulation of the developmental stage of CCs, including their proliferation, differentiation, and apoptosis, was analysed. In particular, for miR-181a-5p, the genes *ACVRIIA* (activin receptor type-2A) and *SMAD3* (small mother against decapentaplegic 3) were considered. Activin, by binding and activating type II receptors (*ACVRIIA)*, promotes granulosa cells’ proliferation and follicle growth and [20], in turn, triggers the Smad signalling pathway through the phosphorylation of SMAD3 and the transcription regulation of its target genes. Hence, the activation of this signalling pathway promotes the proliferation of CCs, repressing their apoptosis, while its inhibition can suppress activin-induced differentiation, causing sub-fertility and generating defects in the regulation and differentiation of granulosa cells [12]. For miR-93-5p, *CDKN1A* (cyclin-dependent kinase inhibitor 1A), encoding a potent cyclin-dependent kinase inhibitor, and *CDK2* (cyclin-dependent kinase 2) were examined. The protein encoded by the *CDKN1A* gene binds and inhibits the activity of kinase 2-cyclin-dependent complexes acting as a regulator of cell cycle progression in the G1 phase. It was demonstrated that miR-93-5p, by suppressing *CDKN1A* expression, promotes cell proliferation, while the reintroduction of *CDKN1A* reverses the proliferative stimulating role of miR-93-5p [21]. *SMAD5* (small mother against decapentaplegic 5), on the other hand, was selected as the target gene of miR-130b-3p. Since SMAD proteins are involved in the proliferation of granulosa and cumulus cells [22], the overexpression of miR-130b-3p promotes the viability and proliferation of granulosa and cumulus cells. Furthermore, it was demonstrated that the inhibition of miR-130b-3p expression during in vitro maturation of oocytes reduces polar globule extrusion [10]. In the case of miRNA let-7g-5p, a target gene involved in the TGF-β/SMAD signalling pathway was analysed. In fact, it was demonstrated that let-7g-5p promotes the apoptosis of granulosa and cumulus cells targeting *TGFβr1* [11], which in turn, via the TGF-β signalling pathway, modulate the regulation of the expression of SMAD proteins [11]. The expression of miR-34a-5p was analysed in relation to its selected target genes, here represented by vascular endothelial growth factor A *(VEGFA)*, *TGFβ1,* and minichromosome maintenance complex component 3 (*MCM3)*. *VEGFA* is a gene that codes for a glycoprotein belonging to the VEGF family, involved both in vasculogenesis and in angiogenesis, two processes that are essential during the luteinization phase. As previously mentioned, TGFβ1 is a multifunctional protein that regulates numerous physiological processes through the activation of the TGF-β signalling pathway, which seems to be, in turn, on the basis of the vasculogenesis process [23]. *MCM3* encodes for the protein maintenance of the mini-chromosome (MCM proteins), necessary for the initiation of the replication process in eukaryotes and the maintenance of genomic integrity [13]. In addition to the miRNAs and target genes mentioned above, miR-202-5p was also analyzed in this study: its target genes identified and analysed in this study are *HAS2* (hyaluronan synthase 2) and *NLRP2* (NLR family pyrin domain contains 2). *HAS2* encodes for an essential enzyme for the synthesis of hyaluronic acid, which represents the structural skeleton of the extracellular matrix of the cumulus and, in turn, is responsible for the expansion of the cumulus oophorus [24]. NLRPs, on the other hand, are members of the NLR (Nod-like receptor) family of proteins. Recently, several NLRPs have been found to be related to reproduction, as several studies on the expression analysis of the NLRP family suggested that many of the genes are expressed specifically in mammalian oocytes [25], and that proteins encoded by them play a crucial role in preimplantation development [26]. Specifically, the NLRP2 inflammasome regulates oocyte quality, and is involved in the decline in fertility associated with age [27]. Furthermore, *NLRP2* has been identified as a maternal effect gene required for early embryonic development in mice. In this regard, the regulation of maternal gene expression resulted in determining the procedural embryonic arrest [28]. Figure 1 schematically displays the miRNAs selected for the study, together with their related genes and the biological processes they modulate.

In addition, in CCs collected from patients treated with rFSH or hMG, Fourier transform infrared microspectroscopy (FTIRM) analysis was applied to evidence the DNA conformational changes that appeared to be related with COS treatments. FTIRM is a vibrational technique, widely applied to investigate the biomolecular building and composition of cells and tissues, allowing one to obtain, in a label-free way, the unique molecular fingerprint of the most relevant biological molecules [29,30]. The spectral profiles obtained by the FTIRM analysis can be correlated with biological processes, such as metabolism, stress status, and apoptosis [31,32,33]. A previous study from our laboratory evidenced the relationship between the metabolic profile of granulosa cells and the fate of the corresponding oocyte, as assessed by gene expression and Fourier transform infrared microspectroscopy (FTIRM). Recently, FTIRM was also exploited to characterize both human oocytes [34] and granulosa cells [22,35], and to assess the effects on human cumulus cells of three controlled ovarian stimulation protocols, based on urinary FSH, recombinant FSH, or hMG [36]. In particular, FTIRM is able to elucidate possible conformational changes in DNA conformation, by evaluating the relative amounts of B-form, A-form, and Z-form DNAs [33]. For example, the B- to A-form DNA transition has been reported to occur, in a reversible way, upon dehydration, possibly as a mechanism to reduce DNA damages [37]. Besides cells’ hydration state, the B- to A-DNA transition also arises, in a non-reversible way, as a response to some drug treatments, especially with compounds interrupting base pairing, like cisplatin [32,38,39]. With regard to Z-form DNA, it is a left-handed conformation, the formation of which is facilitated by DNA methylation; hence, it can be found in specific sequences [40,41].

## 2. Results

### 2.1. Patients’ Data

Table 1 reports patient data related to the response to COS treatments: mean number of retrieved oocytes and percentages of mature and fertilized immature oocytes.

Data displayed, as expected, a higher average number of retrieved oocytes in patients with normal ovarian reserve (NR), with respect to those with low ovarian reserve (LR). Within each group, some differences related to the adopted COS protocol were detectable: first, in the NR group, a significantly higher percentage of fertilized oocytes was found in patients treated with rFSH, with respect to those treated with hMG, while no difference was found with regard to the percentages of mature and immature oocytes; to a further extent, in the LR group, more marked differences due to the stimulation protocol were visible, with the patients treated with hMG displaying significantly higher percentages of mature oocytes and fertilized oocytes.

### 2.2. miRNA Expression and Gene Target Evaluation in CCs

The following miRNAs were considered: miR-181a-5p, miR-93-5p, miR-130b-3p, let-7g-5p, and miR-34a-5p, together with some of their target genes, involved in the regulation of the developmental stage of CCs; the used primer sequences are reported in Table 2 (see Materials and Methods section).

**Figure 2:**
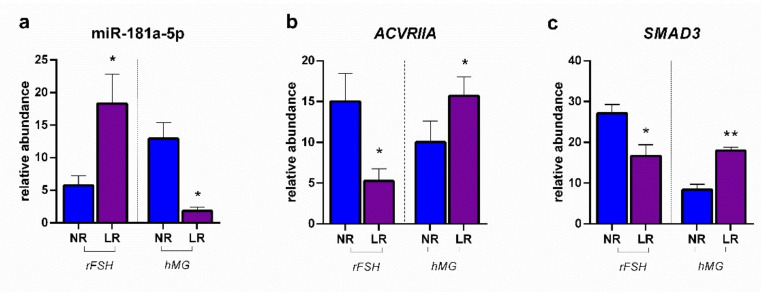
Expression profiles: (**a**) miR-181a-5p; (**b**) *ACVRIIA*: activin A receptor type 2A; (**c**) *SMAD3*: smad family member 3. Results are reported as mean ± standard deviation. Statistical significance was set at *p* < 0.05 and calculated by Student’s *t*-test. Asterisks above columns indicate statistical differences between NR and LR groups. * = *p* < 0.05; ** = *p* < 0.01.

Figure 2 reports the modulation of miR-181a-5p determined in CCs by two different COS treatments patients with normal ovarian reserve (NR) and low ovarian reserve (LR), together with the expression of *ACVRIIA* and *SMAD3*. As the graphs show, ovarian stimulation performed with recombinant FSH determines, in patients with low ovarian reserve, a high expression of the miRNA of interest, which is reflected in a lower expression of the activin IIa receptor; a lower expression of the *SMAD3* gene was also found in LR patients treated with rFSH, with respect to NR ones. With regard to the treatment based on hMG administration, it determined in LR patients a significantly reduced expression of miR-181a-5p, which was reflected in the significantly higher expression of *ACVRIIA*.

**Figure 3:**
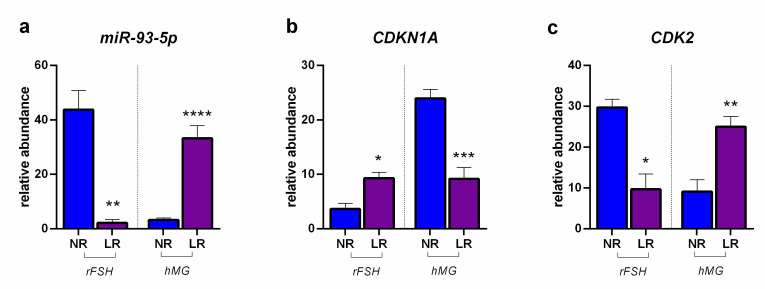
Expression profiles: (**a**) miR-93-5p; (**b**) *CDKN1A*: cyclin dependent kinase inhibitor 1A; (**c**) *CDK2*: cyclin dependent kinase 2. Results are reported as mean ± standard deviation. Statistical significance was set at *p* < 0.05 and calculated by Student’s *t*-test. Asterisks above columns indicate statistical differences between NR and LR groups. * = *p* < 0.05; ** = *p* < 0.01; *** = *p* < 0.001; **** = *p* < 0.0001.

Figure 3 reports the modulation of miR-93-5p determined in CCs by the two examined COS treatments in NR and LR patients, together with the expression of *CDKN1A* and *CDK2*. The results showed a significantly lower expression of miR-93-5p in LR patients treated with rFSH, with respect to NR, which was reflected in a higher expression of the *CDKN1A* target gene, and, subsequently, in a lower expression of *CDK2*, in turn, a target of *CDKN1A*. Conversely, as a response to the COS treatment based on hMG, the expression of miR-93-5p was significantly higher in LR patients with respect to NR, leading to a lower expression of *CDKN1A* and a higher expression of *CDK2*.

**Figure 4:**
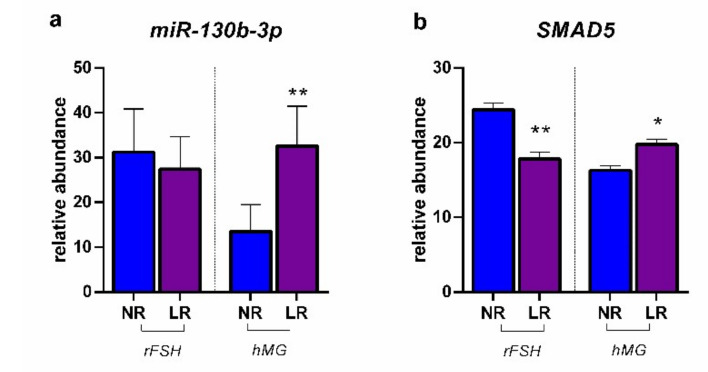
Expression profiles: (**a**) miR-130b-3p; (**b**) *SMAD5*: smad family member 5. Results are reported as mean ± standard deviation. Statistical significance was set at *p* < 0.05 and calculated by Student’s *t*-test. Asterisks above columns indicate statistical differences between NR and LR groups. * = *p* < 0.05; ** = *p* < 0.01.

Figure 4 reports the modulation of miR-130b-3p determined in CCs by the two different COS treatments in NR and LR patients, together with the expression of *SMAD5*. No statistically significant difference was found between NR and LR patients treated with rFSH in terms of miR-130b-3p expression, while the hormonal treatment based on hMG revealed significantly higher values in LR patients. SMAD5 expression showed the same expression profile as miR-130b-3p.

**Figure 5:**
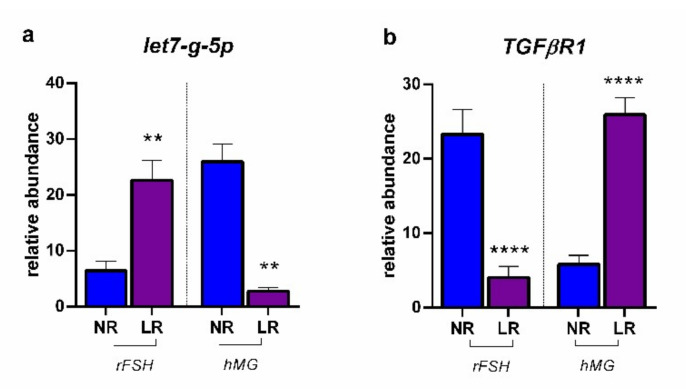
Expression profiles: (**a**) let-7g-5p; (**b**) *TGFβR1*: transforming growth factor beta receptor 1. Results are reported as mean ± standard deviation. Statistical significance was set at *p* < 0.05 and calculated by Student’s t-test. Asterisks above columns indicate statistical differences between NR and LR groups. ** = *p* < 0.01; **** = *p* < 0.0001.

Figure 5 reports the modulation of let-7g-5p in CCs determined by the two different COS treatments in NR and LR patients, together with the expression of *TGFBR1*. The results revealed that the rFSH treatment determined a significantly higher expression of let-7g-5p in LR patients, with respect to NR ones, leading to a significantly lower expression of *TGFBR1*. On the contrary, the administration of hMG resulted in a significantly lower expression of the miRNA of interest in LR patients, consequently leading to the reduced expression of the target gene *TGFBR1*. In both cases, these results are consistent with a high expression of the TGF-β type I receptor, fundamental for the activation of the TGF-β/SMAD signal transduction pathway. *TGFβR1*, in fact, will initiate the intracellular transduction of the signal through the phosphorylation of R-smad, represented, in our case, by *SMAD3* (see Figure 1).

**Figure 6:**
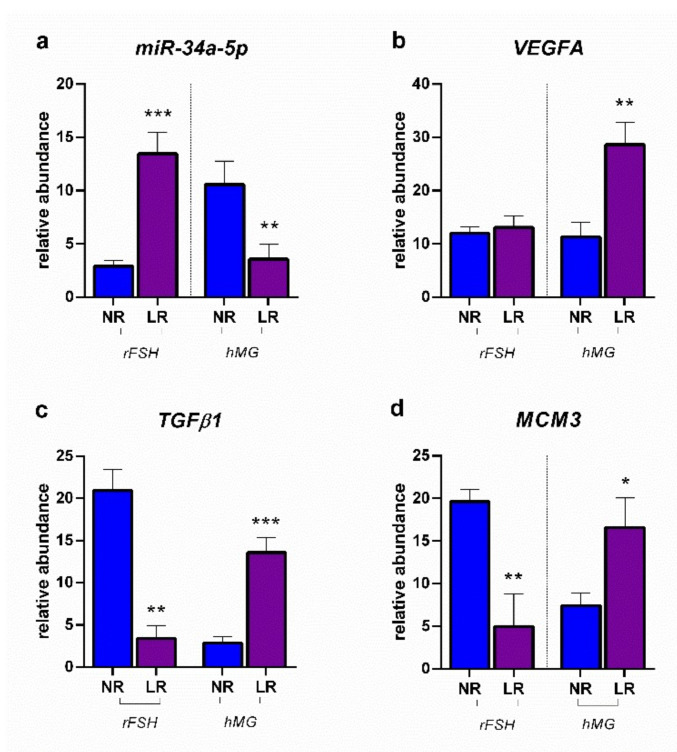
Expression profiles: (**a**) miR-34a-5p; (**b**) *VEGFA*: vascular endothelial growth factor; (**c**) *TGFβ1*: transforming growth factor beta 1; (**d**) *MCM3* minichromosome maintenance complex component 3. Results are reported as mean ± standard deviation. Statistical significance was set at *p* < 0.05 and calculated by Student’s t-test. Asterisks above columns indicate statistical differences between NR and LR groups. * = *p* < 0.05; ** = *p* < 0.01; *** = *p* < 0.001.

Figure 6 reports the modulation of miR-34a-5p determined by the two different COS treatments in NR and LR patients, together with the expression of VEGFA, TGFB1, and MCM3. Data revealed the contrasting response of NR and LR patients to the COS treatment: in fact, the administration of rFSH resulted in significantly higher values of miR-34a-5p expression, consistently leading to a lower expression of TGFB1 and MCM3 (VEGFA showed no significant difference between NR and LR treated with rFSH). The treatment with hMG determined a lower expression of the miRNA of interest in LR patients with respect to NR ones, together with a higher expression of TGFB1, VEGFA, and MCM3.

**Figure 7:**
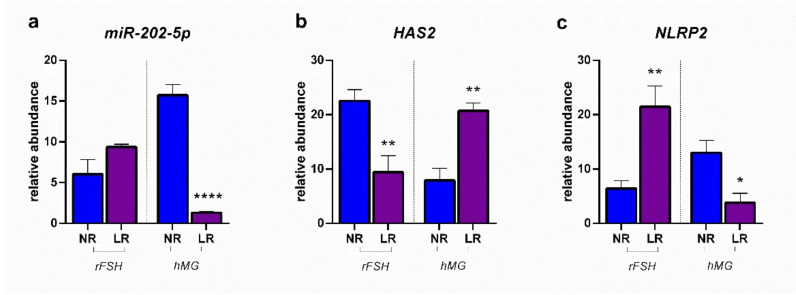
Expression profiles: (**a**) miR-202-5p; (**b**) *HAS2*: hyaluronan synthase; (**c**) *NLRP2:* NLR family pyrin domain containing 2. Results are reported as mean ± standard deviation. Statistical significance was set at *p* < 0.05 and calculated by Student’s t-test. Asterisks above columns indicate statistical differences between NR and LR groups. * = *p* < 0.05; ** = *p* < 0.01; **** = *p* < 0.0001.

Figure 7 reports the modulation of miR-202-5p determined in CCs by the two different COS treatments in NR and LR patients, together with the expression of its target genes, *HAS2* and *NLRP2*. The treatment of poor responders with hMG and normal responders with rFSH showed the inhibition of miR202-5p and *NLRP2* expression, together with an induction of *HAS2* expression.

### 2.3. FTIRM Analysis

CC samples of women treated with rFSH or hMG stimulation protocols were then analyzed by FTIRM, in order to assess the effects of COS treatments on the spectral profile of the nucleic acid component.

**Figure 8:**
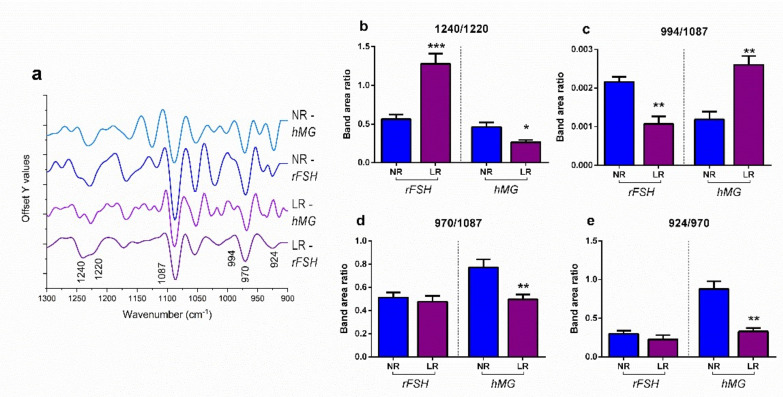
(**a**) Average spectra of NR and LR groups, treated with rFSH and hMG, reported in second derivative mode (1300–900 cm^−1^); the wavenumbers of the most relevant peaks are labelled at the bottom. Results of Student’s *t*-test on band area ratios (**b**) 1240/1220, (**c**) 994/1087, (**d**) 970/1087, and (**e**) 924/970. Histograms show the values of the band area ratios obtained by curve fitting procedure on mean spectra and the mean ± standard deviation spectra of NR and LR groups, treated with rFSH and hMG. Data are reported as mean ± standard deviation. Statistical significance was set at *p* < 0.05 and calculated by Student’s *t*-test. Asterisks above columns indicate a statistically significant difference between NR and LR groups. * = *p* < 0.01; ** = *p* < 0.01; *** = *p* < 0.001.

Figure 8a displays the second derivatives of the average spectra of CCs from NR and LR patients treated with rFSH and NR and LR patients treated with hMG. Second derivatives were exploited to precisely identify the subpeaks of IR bands by means of the curve fitting procedure. The peaks of interest are reported in Table 3 (see Materials and Methods section), in terms of wavenumbers, vibrational modes, and biochemical assignments.

Average absorbance spectra of NR and LR patients treated with rFSH or hMG CC samples and their average ± standard deviation spectra were curve-fitted. Specific band area ratios were calculated and analyzed (Figure 8b–e). Nucleic acids’ relative quantity and characteristics appeared to be affected by the different ovarian stimulation protocols: DNA conformation was affected, in terms of the ratio between the disordered A-form DNA and the ordered B-form DNA (1240/1220 band area ratio), especially in CCs from rFSH-treated LR patients, with respect to NR ones (Figure 8b); the amount of RNA, with respect to total nucleic acids (994/1087 band area ratio), resulted in significantly higher CCs from hMG-treated LR patients and rFSH-treated NR patients (Figure 8c); the amount of double-stranded DNA, with respect to total nucleic acids (970/1087 band area ratio), resulted in being statistically comparable between NR and LR patients treated with rFSH, while significantly higher values were found in CCs from hMG-treated NR patients, with respect to LR ones (Figure 8d). The amount of left-helix DNA (or Z-DNA), with respect to total double-stranded DNA (924/970 band area ratio), resulted in being statistically comparable between NR and LR patients treated with rFSH, while significantly lower values were found in CCs from LR patients treated with hMG, with respect to NR ones (Figure 8e).

## 3. Discussion

In ART practice, the assessment of ovarian reserve is a fundamental aspect to consider in order to select the most appropriate controlled ovarian stimulation protocol and to optimize folliculogenesis. Generally, induction protocols include the employment of FSH, LH, and GnRH analogues with agonist or antagonist action [42]. The choice of stimulation protocols must be characterized based on the benefits or disadvantages that they can bring to the single patient.

The clinical activity of in vitro fertilization is based on the ability of ovarian markers to be able to predict the response to ovarian stimulation. The aim of this study, in fact, is to evaluate the impact of different stimulation protocols on specific miRNAs and their associated target genes, which are known to be important regulators of granulosa and cumulus cells’ function, both in normal reserve (NR) and low reserve (LR) patients.

Among patients treated with rFSH, a significantly higher number and a higher fertilization rate of retrieved oocytes were found in NR patients, with respect to LR ones. Conversely, among patients treated with hMG, a significantly higher percentage of mature and fertilized oocytes was found in LR patients, with respect to NR ones. These results suggest the different response of patients to the hormonal treatments, which can be related to the activation of different molecular pathways.

The present study also evidenced that the two stimulation protocols induced in CCs a differential expression of miR-181a-5p, let-7g-5p, and miR-34-5p, which are involved in the suppression of cell proliferation and thus in follicle apoptosis.

In the present study, ovarian stimulation performed with rFSH determines, in LR patients, a high expression of miR-181a-5p, and a lower expression of the two selected genes *ACVRIIA* and *SMAD3*. These results are in accordance with those previously published, demonstrating that in mice granulosa cells, miR-181a-5p suppresses *ACVRIIA* expression, and in turn modulates granulosa cells proliferation and ovarian follicle development, using Smad2/3 [43]. In addition, rFSH seems to interfere with the activin signaling pathway and with the mechanisms regulating CCs proliferation and follicular growth in LR patients. This is consistent with the lower number of retrieved oocytes with respect to NR. Conversely, NR patients treated with hMG were characterized by a lower expression of miR-181a-5p and a higher expression of *ACVRIIA*, with respect to LR patients.

In the present study, it can be assumed that the treatment with hMG has a positive effect on LR patients, as suggested by the lower expression of miRNA let-7g-5p and the higher expression of the TGF-β type I receptor, responsible for the intracellular transduction of *SMAD3* [11]. Conversely, rFSH is more effective for NR patients, as evidenced by the higher level of both the *TGFβR1* receptor and *SMAD3*.

The expression levels of miR-34-5p, regulating (i) *TGFβ1*, essential for the modulation of CCs proliferation [23], (ii) *VEGFA*, involved in the luteinization process [44] and (iii) *MCM3* crucial in the maintenance of genomic integrity [13], showed that rFSH treatment is the most appropriate for NR patients, while hMG treatment appears to be the most promising in LR patients. Consistent with the results previously obtained, the expression levels of miR-93-5p and miR-130b-3p confirmed that the treatment with rFSH is the most effective for NR patients, while hMG is more suitable for LR patients. In fact, in the present study, a higher expression of miR-93-5p in LR patients treated with hMG and in NR patients treated with rFSH was observed. This result was related to a lower expression of the *CDKN1A* gene that encodes for the kinase inhibitor, and, in turn, a higher expression of the *CDK2* gene involved in the progression of the cell cycle [21]. No significant change in miR-130b-3p expression was found between NR and LR patients treated with rFSH. On the contrary, miR-130b-3p levels were higher in LR patients with respect to NR treated with hMG. *SMAD5* involved in promotion of CCs proliferation, selected as the target gene of miR-130b-3p, showed a similar expression profile in both groups after rFSH and hMG treatments. These results evidenced that rFSH is more efficient in NR patients, while hMG performs better in LR patients.

In addition to the miRNAs and target genes mentioned above, miR-202-5p and its target genes *HAS2* (hyaluronan synthase 2) and *NLRP2* (Nod-like receptor family pyrin domain contains 2) were analyzed. miR-202-5p showed a higher expression level in LR patients treated with rFSH, with respect to NR ones, and in NR patients treated with hMG, with respect to LR ones. The subsequent expression profiles of *HAS2* and *NLRP2*, respectively, positively and negatively correlated with oocyte quality, confirm the positive effect of the rFSH treatment on NR patients, and of hMG on LR patients.

A vibrational analysis of nucleic acids’ relative quantity and conformation was performed by FTIR microspectroscopy. The high transcriptional activity suggested by the analysis of 994/1087 band area ratio in LR patients treated with hMG and NR ones treated with rFSH, indicated a higher proliferation rate of CCs, with respect to LR patients treated with rFSH, and NR patients treated with hMG. The low level of RNA in these two latter groups seemed to be caused by two different mechanisms: first, the LR patients treated with rFSH displayed the highest values of the 1240/1220 band area ratio, which indicates high amounts of the unordered A-form DNA, which is less efficiently transcribed [32,38,39]; second, in NR patients treated with hMG, the lower transcriptional levels may be associated with the highest levels of the 924/970 band area ratio, representative of the Z-DNA amount. Z-DNA is a left-handed conformation of DNA, often found in specialized sequences as a result of DNA methylation processes, which strongly influences the transcriptional activity via the exclusion of transcription factors [33,40,41,45]. Hence, the different stimulation treatment in ART patients with normal and low ovarian reserve seems to determine a change in the conformational state of the CCs DNA; in particular, the different order (A-DNA and B-DNA) and the methylation state (Z-DNA) [33,37,46], representing the real novelty of this study.

## 4. Materials and Methods

### 4.1. Ethical Approval

The study, approved by the ethics committee n. 2020241, was carried out in full accordance with ethical principles for experiments involving humans, including The Code of Ethics of the World Medical Association (Declaration of Helsinki, 2013). Patients participating in the investigation signed a written informed consent agreement, which included the donation of CCs. All samples were strictly anonymous, making it impossible to correlate them to patients.

### 4.2. Patient Enrolment and Experimental Design

A cohort of 51 women was enrolled in an IVF program, according to specific inclusion criteria: women with idiopathic infertility and/or tubal infertility; Mediterranean diet, no smoking, no consumption of alcohol, and no consumption of drugs. Exclusion criteria for the cohort selection were: hyper responders, pelvic endometriosis, previous ovarian surgery, presence of ovarian masses, pelvic inflammatory disease, autoimmune or chronic systemic diseases. Women were divided into two groups, according to their serum levels of anti-Müllerian hormone (AMH): patients with normal ovarian reserve (NR; n. 36; age 36.25 ± 3.09) had AMH levels of 1.2–4.0 ng/mL [18], while patients with low ovarian reserve (LR; n. 15; age 40.77 ± 2.62) had AMH levels <1.2 ng/mL [17].

After patients’ selection and division into NR and LR groups, two different follicle-stimulated gonadotropins were administered:

1. Twenty-seven patients (9 LR and 18 NR) treated with rFSH formulation → Follitropin α and β: these are recombinant molecules of the natural human follicle stimulating hormone (Puregon^®^, MSD, Roma, Italy; Gonal-F^®^, Merck, Darmstadt, Germany; Ovaleap^®^, Theramex, London, UK).
2. Twenty-four patients (6 LR and 18 NR) treated with FSH + LH formulation → Menotropin (hMG): is a highly purified preparation of FSH and LH in a 1:1 ratio, extracted from the urine of postmenopausal women (Meropur^®^, Ferring, Sweden).

For all the experimental groups, the short protocol of gonadotropin COS was used (daily subcutaneous injection started on the second day of the menstrual cycle), GnRH antagonist (Cetrotide, Merck, Darmstadt, Germany; Orgalutran, MSD, Roma, Italy) was administered on initiation by a follicle of 12–14mm, and HCG 10.000 UI was used for ovulation triggering 36 h before oocyte retrieval. The gonadotropin doses for patients included in the study were statistically comparable. Moreover, during COS, an ultrasound transvaginal scan (DC-70, Mindray Medical Italy S.R.L., Italy) for follicular growth monitoring was performed.

### 4.3. CCs Samples Collection and Preparation

Oocyte collection was carried out under echographic control, with the use of a needle (Ovum aspiration needle 37 ga–35 cm, COOK Medical, Bloomington, IN, USA) filled with buffered medium (Flushing Medium with Heparin 10 IU/mL) and applied to a transvaginal probe, in order to aspirate follicular fluid and retrieve oocytes. Follicular fluid was recovered in a test tube, 37 °C—maintained, and then poured in a Petri dish (Thermo Fisher Scientific Inc., Waltham, MA, USA) for visualization by a stereomicroscope (Stemi 508, provided with a Thermo plate; ZEISS, Oberkochen, Germany), to identify the cumulus and oocyte cells’ complexes (COCs). COCs were washed with a buffered medium at 37 °C (Flushing Medium, Origio, CooperSurgical, Denmark) and subsequently transferred into an incubator, and into a culture dish (Thermo Fisher Scientific Inc., Waltham, MA, USA) with an unbuffered and pre-equilibrated culture medium at 37 °C.

In order to remove CCs and denudate oocytes, one of the four wells of the IVF-4-Well Dish (Thermo Fisher Scientific Inc., USA) was filled with 150 µL of pre-warmed (37 °C) buffered culture medium (Flushing Medium, Origio, CooperSurgical, Målov, Denmark) and 150 µL of pre-warmed (37 °C) SynVitro Hyadase (Origio, CooperSurgical, Målov, Denmark); the other three wells were filled with only pre-warmed (37 °C) buffered culture medium. COCs were placed in the well with SynVitro Hyadase and gently aspirated up and down by a denuding pipette (Flexipet^®^ denuding pipette, COOK Medical, Bloomington, IN, USA). Oocytes were transferred into a well rinsed with culture medium, and the remaining cumulus cells were removed. Oocytes were assessed for nuclear maturity: oocytes with the extruded first polar body were considered mature (metaphase II, MII); both oocytes at the germinal vesicle (GV) stage and metaphase I (MI) stage were generally taken into account as “immature”. In order to remove the culture medium and prepare cells for further analyses, CC samples were centrifuged at 1000× *g* for 15 min and washed using physiologic salt solution (Merck, Germany); the procedure has been repeated twice. The final CCs pellet was resuspended in 500 µL of a physiologic salt solution, and divided according to the subsequent analyses: 10 µL of the cell suspension was fixed in a 4% paraformaldehyde (PFA) solution for 10 min, washed twice in a physiological salt solution, and then stored at 4 °C until FTIR measurements; the remaining cells were centrifuged, and the pellet was stored at −80 °C until gene expression analysis.

### 4.4. Total RNA Extraction and cDNA Synthesis

From each CCs sample, small (<200 bp) and total (>200 bp) RNA fractions were extracted using the miRNeasy Mini Kit^®^ (Qiagen, Hilden, Germany) extraction kit, following the manufacturer’s protocol. Total RNA extracts were eluted in 30 µL of RNAse-free water. Final RNA concentrations were determined with a NanoDrop™ 1000 Spectrophotometer (Thermo Fisher Scientific Inc., Waltham, MA, USA), and RNA integrity was verified by Gel Red (Biotium Inc., Fremont, CA, USA) staining of 28S and 18S ribosomal RNA fragments on 1% agarose gel. RNA was stored at −80 °C until use. Total RNA was treated with DNAse (10 IU at 37 °C for 10 min, Thermo Fisher Scientific Inc., Waltham, MA, USA). An aliquot of 100 ng of each RNA sample was polyadenylated with *E.coli* poly(A) polymerase (New England Biolabs) and converted into cDNA with the SuperScript-II cDNA Synthesis Kit (Invitrogen, Thermo Fisher Scientific Inc., Waltham, MA, USA), primed with an oligo dT adaptor [47].

### 4.5. Real-time Polymerase Chain Reaction

For each selected miRNA sequence, the primers couple was selected as follows: the forward primer spanned the entire mature sequence and the reverse universal primer was complementary to the poly(T) adaptor [47]. The relative quantification of gene expression was performed with the SYBR Green method in a CFX96 Real-Time PCR system (Bio-Rad, Hercules, CA, USA). PCR was carried out in duplicate for each analyzed sample. Reactions were set by mixing 1 µL diluted (1/10) cDNA, 5 µL of 2× SYBR Green PCR Master Mix (Bio-Rad, USA), containing SYBR Green as a fluorescent intercalating agent, 0.2 µM of forward and reverse primers, and 3.8 µL of Milli-Q water. The thermal profile was as follows: enzyme activation at 95 °C for 3 min; 45 cycles of denaturation (10 s at 95 °C), followed by 20-s annealing at 60 °C for all the examined miRNAs and genes, and 20-s elongation at 72 °C. Fluorescence was monitored at the end of each cycle. Dissociation curves for primer specificity and absence of primer–dimer formation check were performed and consistently showed a single peak. Ribosomal protein L13a (*RPL13A*) and β-actin (*ACTB*) were used as internal controls to enable result standardization by eliminating variations in mRNA and cDNA quantity and quality [48]. These genes were chosen because their mRNA levels did not vary between experimental groups. No amplification products were observed in negative controls and no primer-dimer formations were observed in the control templates. Data were analyzed using CFX Manager Software version 3.1 (Bio-Rad, USA). The quantification method was based on a ∆∆Ct calculation implemented with the Pfaffl equation, to improve accuracy by accounting for varied reaction efficiencies depending on primers [49,50]. The modification of gene expression is represented with respect to zero. The target genes for each miRNA of interest were selected by means of the DIANA-TarBase v8 software [51]. The used primer sequences are reported in Table 2.

### 4.6. FTIRM Analysis

FTIRM measurements were performed at the Laboratory of Advanced Research Instrumentation, Department of Life and Environmental Sciences, Università Politecnica delle Marche (Ancona, Italy). A Hyperion 3000 Vis-IR microscope equipped with a HgCdTe (MCT_A) detector and coupled with an INVENIO interferometer (Bruker Optics, Ettlingen, Germany) was used.

An aliquot of 10 µL of CCs from each patient was deposited onto CaF_2_ optical windows (1-mm thick, 13-mm diameter) and air-dried for 30 min, in order to avoid water contributions to the IR spectra [22,35]. From each aliquot of CCs sample, ~10 microareas (30 × 30 μm^2^) containing densely packed cell monolayers were selected by visible microscopy, on which IR spectra were collected in the transmission mode in the medium infrared region (4000–800 cm^−1^), averaging 512 scans, with a spectral resolution of 4 cm^−1^, a zero-filling factor of 2, and a scanner velocity of 40 kHz. Background spectra were collected using the same parameters on clean zones of the CaF_2_ optical windows. All raw IR spectra underwent the following pre-processing procedure: the spectral contributions of water were subtracted by using an in-house optimized routine [52]; then, subtracted spectra were corrected for the contribution of atmospheric carbon dioxide and water vapor (Atmospheric compensation routine, OPUS 7.1 software, Bruker Optics, Ettlingen, Germany) and vector normalized in the entire spectral range (Normalization routine, OPUS 7.1 software). Subsequently, spectra were evaluated based on the height of the band centered at ~1660 cm^−1^ (Amide I band of proteins), which is always the highest peak of cell spectra; average spectra with, at 1660 cm^−1^, absorbance values lower than 0.07 a.u. (~20%), were discarded [31]. On these pre-processed spectra, for each patient, the average absorbance spectrum and its corresponding ± standard deviation spectra were calculated. After the calculation of second derivates (Savitzky–Golay filter, 13 points of smoothing), these spectra were curve-fitted in the 1300–900 cm^−1^ region, representative of nucleic acids. The selected spectral region was fitted with Gaussian band components upon straight baseline correction and vector normalization (GRAMS/AI 7.02, Galactic Industries, Inc., Salem, NH, USA). The underlying bands were precisely identified by second derivative results, and their center values (expressed as wavenumbers), together with the corresponding vibrational mode and biochemical assignment, are reported in Table 3.

The integrated areas of selected underlying bands were calculated and used to obtain the following band area ratios: 1240/1220 (representing the relation between A-form and B-form DNA); 994/1087 (representing the RNA amount with respect to nucleic acids); 970/1087 (representing the double-strand DNA amount respect to nucleic acids), and 924/970 (representing the left-handed Z-DNA component of double-stranded DNA).

## 5. Conclusions

The identification of informative biomarkers is the key to understanding pathophysiological processes and represents the next crucial challenge for the development of new therapeutic strategies. The results here presented clearly evidenced how different COS protocols modulate the expression of key genes for cumulus cells’ function, with different effects according to the ovarian reserve of patients. Moreover, the COS protocols demonstrated that it determines a different conformation and methylation state of cumulus cells’ DNA, which resulted in being related to the expression of miRNAs and their target genes analyzed in this study. In particular, the results here presented showed that the treatment with rFSH is the most appropriate in patients with normal ovarian reserves, while the treatment with hMG appears to be the most suitable in patients with low ovarian reserves. These favorable effects are related to the positive control of the expression of genes involved in follicular cells’ proliferation, vasculogenesis, angiogenesis, genomic integrity, and oocyte quality.

Based on this knowledge, we firmly believe that a better understanding of the role of miRNAs and their modulation by hormonal treatment can be an important step in improving female reproductive health.

## Figures and Tables

**Table 1:** Summary of patients’ data: number of average retrieved oocytes, percentage of immature, mature, and fertilized oocytes. Data of number of retrieved oocytes are reported as mean ± standard deviation. Different letters indicate a statistically significant difference (*p* < 0.05) and are calculated by ANOVA followed by Tukey’s multiple comparisons test (average retrieved oocytes) and by Chi square test (% immature, % mature oocytes, and % fertilized oocytes).

Treatment	# Patients	Ovarian Reserve	Retrieved Oocytes	% Immature Oocytes	% Mature Oocytes	% Fertilized Oocytes
*rFSH*	9	low	3.44 ± 1.88 ^a^	21.00% ^a^	79.00% ^a^	69.45% ^a^
	18	normal	8.06 ± 4.22 ^b^	22.06% ^a^	78.00% ^a^	78.64% ^b^
*hMG*	6	low	3.00 ± 1.41 ^a^	8.83% ^b^	91.17% ^b^	91.67% ^c^
	18	normal	6.33 ± 3.70 ^ab^	28.72% ^a^	71.28% ^a^	70.00% ^a^

**Table 2:** List of primers used for miRNA and gene expression analyses by real-time qPCR (*ACTB*: actin beta; *RPL13A*: ribosomal protein L13a; *ACVRIIA*: activin A receptor type 2A; *SMAD3*: SMAD family member 3; *CDKN1A*: cyclin dependent kinase inhibitor 1A; *CDK2*: cyclin dependent kinase 2; *HAS2*: hyaluronan synthase 2; *NLRP2*: NLR family pyrin domain containing 2; *SMAD5*: SMAD family member 5; *TGFBR1*: transforming growth factor beta receptor 1; *VEGFA*: vascular endothelial growth factor A; *TGFB1*: transforming growth factor beta 1).

miRNA	Forward Primer (5′–3′)	Universal Reverse Primer (5′–3′)	miRBase Accession Number
*miR-181a-5p*	AACATTCAACGCTGTCGGTGAGT	GCGAGCACAGAATTAATACG	MI0000269
*miR-93-5p*	CAAAGTGCTGTTCGTGCAGGTAG	GCGAGCACAGAATTAATACG	MI0000095
*miR-23a-5p*	GGGGTTCCTGGGGATGGGATTT	GCGAGCACAGAATTAATACG	MI0000079
*miR-202-5p*	TTCCTATGCATATACTTCTTTG	GCGAGCACAGAATTAATACG	MI0003130
*miR-130b-3p*	CAGTGCAATGATGAAAGGGCAT	GCGAGCACAGAATTAATACG	MI0000748
*let-7g-5p*	TGAGGTAGTAGTTTGTACAGTT	GCGAGCACAGAATTAATACG	MI0000433
*miR-34a-5p*	TGGCAGTGTCTTAGCTGGTTGT	GCGAGCACAGAATTAATACG	MI0000268
**Gene**	**Forward Primer (5′–3′)**	**Reverse Primer (5′–3′)**	**NCBI Accession Number**
*ACTB*	GCAGAAGGAGATCACATCCCTGGC	CATTGCCGTCACCTTCACCGTTC	NM_001101.5
*RPL13A*	TCTGGAGGACTGTAAGAGGTATGC	AGACGCACAATCTTGAGAGCAG	NM_012423.3
*ACVRIIA*	CCTCGGACTTTAGGTGTCTGG	CCAAGTATAGCACCTGAAGAACA	NM_001278579.2
*SMAD3*	GCCTGTGCTGGAACATCATC	TTGCCCTCATGTGTGCTCTT	NM_005902.4
*CDKN1A*	AGGTGGACCTGGAGACTCTCAG	TCCTCTTGGAGAAGATCAGCCG	NM_000389.5
*CDK2*	ATGGATGCCTCTGCTCTCACTG	CCCGATGAGAATGGCAGAAAGC	NM_001798.5
*HAS2*	GTCATGTACACAGCCTTCAGAGC	ACAGATGAGGCTGGGTCAAGCA	NM_005328.3
*NLRP2*	CATTCTGCGTCAAGCACTGTCG	CCGTCCAGAAAGGAAGCATGTG	NM_001348003.2
*SMAD5*	CAGGAGTTTGCTCAGCTTCTGG	GGTGCTGGTTACATCCTGCCG	NM_005903.7
*TGFBR1*	GACAACGTCAGGTTCTGGCTCA	CCGCCACTTTCCTCTCCAAACT	NM_004612.4
*VEGFA*	TTGCCTTGCTGCTCTACCTCCA	GATGGCAGTAGCTGCGCTGATA	NM_001025366.3
*TGFB1*	GCCGTGGAGGGGAAATTGAG	TGAACCCGTTGATGTCCACTT	NM_000660.7
*MCM3*	CGAGACCTAGAAAATGGCAGCC	GCAGTGCAAAGCACATACCGCA	NM_002388.6

**Table 3:** Center position (wavenumbers), vibrational mode, and biochemical assignment of the underlying bands as identified by curve fitting analysis of average absorbance spectra of the experimental groups in the 1300–900 cm^−1^ spectral range.

Wavenumber	Vibrational Mode and Biochemical Assignment	References
~1240	Asymmetric stretching vibrations of phosphate moieties of A-form DNA	[32,37,38,53]
~1220	Asymmetric stretching vibrations of phosphate moieties of B-form DNA	[32,37,38,53]
~1087	Symmetric stretching vibrations of phosphate moieties in nucleic acids	[54,55,56]
~994	C–C, C–O ring breathing of RNA ribose	[31,32,54,57]
~970	Backbone vibrations of nucleic acids, mainly double-strand DNA	[32,35,58]
~924	Left-handed helix DNA vibrations (Z-DNA)	[33,46,58,59]

## Data Availability

Data are contained within the article.
